# Comparison of Endemic and Epidemic Vesicular Stomatitis Virus Lineages in *Culicoides sonorensis* Midges

**DOI:** 10.3390/v14061221

**Published:** 2022-06-03

**Authors:** Paula Rozo-Lopez, Steven J. Pauszek, Lauro Velazquez-Salinas, Luis L. Rodriguez, Yoonseong Park, Barbara S. Drolet

**Affiliations:** 1 Department of Entomology, Kansas State University, Manhattan, KS 66506, USA; paularozo@ksu.edu (P.R.-L.); ypark@ksu.edu (Y.P.); 2 Foreign Animal Disease Diagnostic Laboratory, Plum Island Animal Disease Center, Animal and Plant Health Inspection Service, United States Department of Agriculture, Orient Point, NY 11957, USA; steve.pauszek@usda.gov; 3 Foreign Animal Disease Research Unit, Plum Island Animal Disease Center, Agricultural Research Service, United States Department of Agriculture, Orient Point, NY 11957, USA; lauro.velazquez@usda.gov (L.V.-S.); luis.rodriguez@usda.gov (L.L.R.); 4 Arthropod-Borne Animal Diseases Research Unit, Center for Grain and Animal Health, Agricultural Research Service, United States Department of Agriculture, Manhattan, KS 66502, USA

**Keywords:** vesicular stomatitis virus, *Culicoides* midges, viral lineages, vector competence

## Abstract

Vesicular stomatitis virus (VSV) primarily infects livestock and is transmitted by direct contact and vectored by *Culicoides* midges (Diptera: Ceratopogonidae). Endemic to Central and South America, specific VSV lineages spread northward out of endemic regions of Mexico and into the U.S. sporadically every five to ten years. In 2012, a monophyletic epidemic lineage 1.1 successfully spread northward into the U.S. In contrast, the closest endemic ancestor, lineage 1.2, remained circulating exclusively in endemic regions in Mexico. It is not clear what roles virus-animal interactions and/or virus-vector interactions play in the ability of specific viral lineages to escape endemic regions in Mexico and successfully cause outbreaks in the U.S., nor the genetic basis for such incursions. Whole-genome sequencing of epidemic VSV 1.1 and endemic VSV 1.2 revealed significant differences in just seven amino acids. Previous studies in swine showed that VSV 1.1 was more virulent than VSV 1.2. Here, we compared the efficiency of these two viral lineages to infect the vector *Culicoides sonorensis* (Wirth and Jones) and disseminate to salivary glands for subsequent transmission. Our results showed that midges orally infected with the epidemic VSV 1.1 lineage had significantly higher infection dissemination rates compared to those infected with the endemic VSV 1.2 lineage. Thus, in addition to affecting virus-animal interactions, as seen with higher virulence in pigs, small genetic changes may also affect virus-vector interactions, contributing to the ability of specific viral lineages to escape endemic regions via vector-borne transmission.

## 1. Introduction

Vesicular stomatitis (VS) is a viral disease caused by a Rhabdovirus that infects cattle, horses, and swine [1]. VS viruses (VSV) are classified by serotypes, Indiana (VSV-IN) and New Jersey (VSV-NJ), based on the distinct neutralizing antibodies generated in infected animals [2,3]. Clinical signs include vesicular lesions in the gums, tongue, oro-nasal mucosa, teats, and coronary bands [4,5]. Infection with VSV negatively impacts animal health and livestock production, causing economic losses and supply chain interruptions [1]. Additionally, quarantines and trade restrictions are imposed on affected premises due to the clinical resemblance of foot-and-mouth disease in cattle and swine [6].

VS is endemic in tropical and subtropical regions of the Americas [7]. In the U.S., where VS is not endemic, outbreaks display an occurrence pattern of five to ten-year intervals [6]. Historically, VS outbreaks initiated in the Mexico-U.S. border and spread as far north as Wyoming, lasting for a single year (incursion year), or often re-emerging for a second year (expansion year) [8,9,10]. These epidemic viruses are associated with a distinct viral lineage, closely related to endemic lineages circulating in Mexico [8,10,11]. In 2012, a specific VSV-NJ lineage, 1.1, spread northward through central and northern Mexico and made an incursion into the southern U.S. Phylogenetic characterization suggested that the closest common endemic ancestor of lineage 1.1 was lineage 1.2, a group of viruses confined in the endemic area in Veracruz, Mexico [11]. Further studies indicated that genetic differences between the epidemic (1.1) and endemic (1.2) lineages included 111 nucleotide substitutions associated with 23 amino acid changes in four out of the five viral proteins encoded by VSV [12]. These amino acid changes were classified mostly as favorable or neutral; however, seven non-synonymous substitutions between the two lineages were predicted to significantly affect protein size, charge, or hydrophobicity [12]. Although the specific impact of these amino acid mutations is still unclear, additional evolutionary studies have suggested a potential relevance during the evolution of VSV strains [13,14] and a role in mediating the immune response in the mammalian host [15,16,17,18]. Previous comparative infection studies in swine showed that the epidemic lineage 1.1 had an increased ability to disrupt innate immune responses, caused higher fever, and presented with an increased number of vesicular lesions compared with infections using the closely related endemic lineage 1.2 [12,19].

VSV epidemiology is complex. In addition to virus being transmitted between several mammalian host species through direct contact and fomites, multiple insect species play a role in transmission as biological and mechanical vectors [20]. Among the main biological vector species, *Culicoides sonorensis* (Diptera: Ceratopogonidae) Wirth and Jones (1957) is one of the most common biting midge species associated with livestock across the U.S. [21,22]. Adult *Culicoides* females opportunistically pool-feed on the blood of a wide range of hosts every 3 to 5 days to obtain protein for egg-laying [23]. VSV oral acquisition occurs when midges pool-feed on VSV-infected animals near vesicular lesions or on skin surfaces contaminated with saliva containing high virus titers [24]. Ingested virus particles must then survive the digestive environment of the midgut while infecting and replicating in the midgut epithelium. VSV progeny virions must escape the midgut, disseminate into the hemolymph, and infect secondary tissues. The transmission cycle is completed when VSV reaches the vector’s salivary glands to be released into the saliva during subsequent blood-feeding on susceptible hosts [25].

Successful arbovirus biological transmission of any viral lineage implies that the virus overcomes infection barriers in the vector in addition to immune and transcriptional responses specific to virus genotypes [26]. The midgut is the most critical organ in determining vector competence [27]. To infect the midgut epithelium, a threshold level of VSV infective particles in a blood meal is required [28]. However, this infection threshold can be different for specific virus and vector populations [27]. Additional physical barriers (i.e., the peritrophic matrix and viral entry into the midgut cells) and antiviral immune responses [i.e., RNA interference (RNAi), Janus kinase signal transducer (JAK-STAT), and Toll pathway] may also limit successful midgut infection [26,29]. Thus, when a vector is refractory to a specific arbovirus, a midgut infection barrier is first hypothesized [27,29].

Following replication in the midgut, arboviruses must disseminate through the midgut basal lamina into the hemolymph to reach secondary tissues (i.e., fat bodies, reproductive tissues, and ultimately the salivary glands). The ability or inability of viruses to disseminate from the midgut is mainly based on viral genetics but may also be related to the infectious dose in the blood meal [30]. To succumb to the midgut escape barrier implies that the arboviruses fail to successfully disseminate and replicate throughout secondary tissues despite midgut infections, thus minimizing transmission [27,29,31].As with other aspects of vector competence, successful virus transmission via subsequent blood-feeding is also virus-vector species-specific [27]. However, a salivary gland escape barrier, where the virus is present in the salivary glands but is inefficiently transmitted, has not been reported in *Culicoides* [32,33].

The importance of *C. sonorensis* as a VSV vector has been well established [25,34,35,36,37]. Previous research has shown a detailed description of the vector competence and the temporal progression of VSV infection in the midge [25,28,34], bite transmission [35,36], non-conventional routes of transmission [37], and vector capacity variation [38]. However, the influence of viral genetic factors on *Culicoides*-virus interactions and their potential impact on transmission are understudied. To provide insight into genetic determinants of VSV vector-borne transmission and the potential role vector-virus interactions play in the ability of a specific viral lineage to expand as an epidemic virus into the U.S., we evaluated whether the small genetic changes in epidemic (1.1) and endemic (1.2) VSV lineages associated with increased virulence in pigs [12] also affected vector-virus interactions. We compared the ability of both lineages to infect the midgut and disseminate into the salivary glands of the VSV vector *C. sonorensis*.

## 2. Materials and Methods

### 2.1. Vesicular Stomatitis Viral Lineages

The VSV-NJ strains NJ0612NME6 and NJ0806VCB were used in this study to represent the epidemic lineage 1.1 and its closest endemic ancestor lineage 1.2, respectively [11]. These viruses were recovered using a cDNA clone system as previously published [39]. In the case of strain NJ0806VCB the plasmid LC-KAN-NJ0806VCB containing the full-length genome of the endemic strain was synthesized (Epoch Life Sciences, Sugar Land, TX, USA). Independent co-transfections of either LC-KAN-NJ0612NME6 or LC-KAN-NJ0806VCB along with the supporting plasmids P-TIT-VSV-N, P-TIT-VSV-P and P-TIT-VSV-L were conducted on BSR-T7/5 cells [40]. The presence of rNJ0612NME6 and rNJ0806VCB was confirmed from DNAse I treated supernatant using RT-qPCR as previously described [12]. Viral stocks of each virus were produced in BHK-21 cells, and final titers were determined by TCID_50_/mL using BHK−21 cells. Next-generation sequencing (NGS) was performed as previously described to verify 100% sequence identity between rNJ0612NME or rNJ0806VCB and their parental viruses [41,42].

### 2.2. Cell Lines

Porcine epithelial cells (AG08113; Coriell Institute, Camden, NJ, USA) were maintained in Eagles MEM with Earle’s salts (Sigma, St. Louis, MO, USA) containing 2% FBS and 100 U penicillin/streptomycin sulfate at 37 °C with 5% CO_2_. *Culicoides* cells [W8; USDA, Arthropod-Borne Animal Diseases Research Unit (ABADRU), Manhattan, KS, USA] were maintained in Schneider’s Insect Media (Sigma-Aldrich, St. Louis, MO, USA) (24.5 g/L) supplemented with 0.4 g/L sodium bicarbonate, 0.0585 g/L L-glutamine, 0.006 g/L reduced glutathione, 0.03 g/L L-asparagine, 18 μL of 10 mg/L bovine insulin and 5% FBS at 28 °C with a CO_2_ concentration of 0.2%. Vero MARU cells (VM; Middle America Research Unit, Panama) grown in 199E media (Sigma-Aldrich, St. Louis, MO, USA) containing 2% FBS, 100 ug/mL of streptomycin, 100 U/mL of penicillin, and 0.25 ug/mL of amphotericin B at 37 °C with 5% CO_2_ were used for detecting and quantitating infectious virus from midge samples as described below.

### 2.3. In Vitro Growth

**Figure 1:**
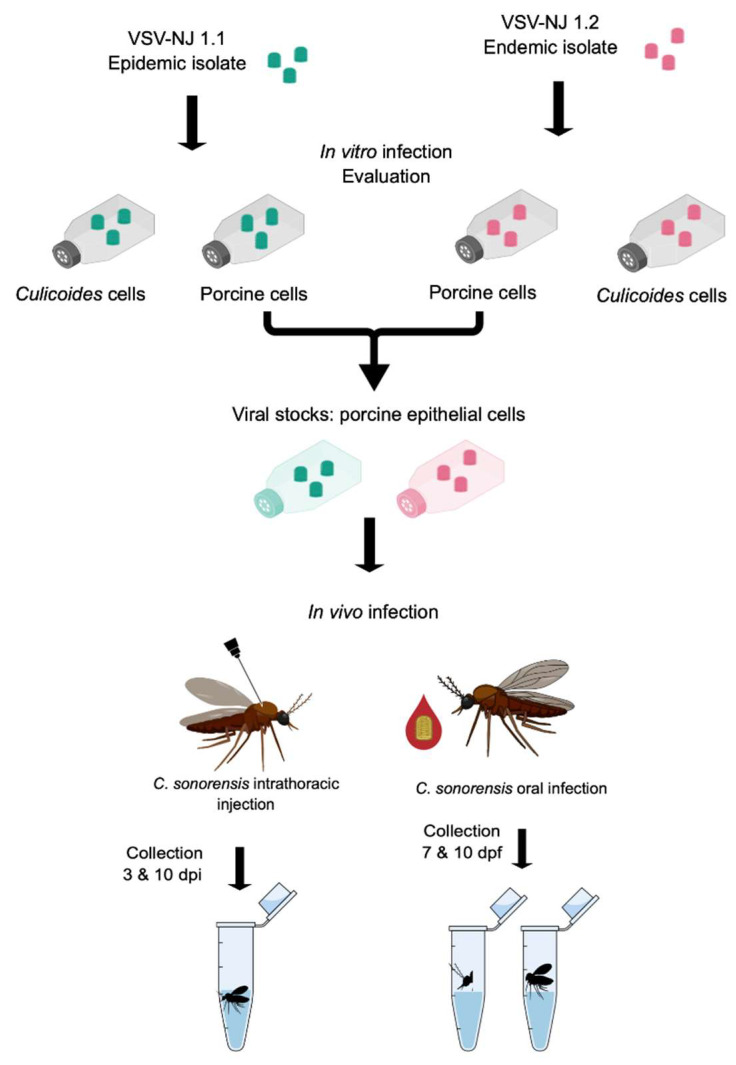
Experimental design to evaluate the recombinant VSV epidemic lineage 1.1 (rNJ0612NME6; teal) and the endemic lineage 1.2 (rNJ0806VCB; pink) with *in vitro* growth in porcine epithelial and *Culicoides* cells, and subsequent *in vivo* infection of *Culicoides sonorensis* midges after intrathoracic injection (whole bodies tested) and oral infection (heads/salivary glands tested for dissemination and bodies tested for midgut infection).

To study the growth kinetics of rNJ0612NME6 (1.1) and rNJ0806VCB (1.2) in porcine epithelial and *Culicoides* cells (Figure 1), confluent cells in T25 flasks were washed twice with phosphate-buffered saline (PBS). Flasks were then infected at an MOI of 0.1 with each virus lineage originally produced in BHK-21 cells. Flasks were incubated with the virus inoculum and rocked every 20 min. After one hour, 4 mL of maintenance media was added, and all flasks were returned to the incubators. At each sampling time (0, 12, 24, 36, 48, 72, 96, 120, 144, and 168 h post-infection; hpi), a flask of each cell type was stored at –80 °C until further processing.

Virus was harvested by performing two freeze/thaw cycles and then clearing the supernatant by centrifugation (1500× *g* for 10 min at 4 °C). Aliquots of cleared supernatants were stored at −80 °C. Virus was titered as below using 100 μL of the cleared supernatants in a standard plaque assay on Vero cells. Total RNA was extracted using 500 μL of the cleared supernatants and evaluated by RT-qPCR (described below). An additional 50 μL of total RNA samples were submitted to the Kansas State Veterinary Diagnostic Laboratory for Illumina next-generation deep sequencing of the whole VSV genome to confirm 100% sequence identity with the parental inoculum viruses. In addition to growth kinetics, both viral lineages were propagated (as detailed above) at an MOI of 0.01 in porcine epithelial cells to produce high titer viral stocks for subsequent *Culicoides* midge infection studies (Figure 1). All virus stocks were stored at −80 °C.

### 2.4. In Vivo Infection of Culicoides sonorensis Midges

Adult *C. sonorensis* midges from the AK colony (USDA, ABADRU, Manhattan, KS, USA) [43] were used for all experiments. The AK colony was established in 1973 and has been continuously produced for approximately 700 generations without deleterious inbreeding effects [44]. To assess viral replication in midges without a midgut barrier, newly emerged females (1–3 days post-emergence) were anesthetized with CO_2_ and intrathoracically injected with 60 nL of a virus lineage propagated in porcine epithelial cells (Figure 1) [37]. All intrathoracic injections were performed with porcine cell-derived viral stocks at a titer of 6.4 log_10_ PFU/mL.

To assess the ability of each lineage to infect and escape the midgut barrier to disseminate to salivary glands, newly emerged females were allowed to feed on a glass water-jacketed bell feeder (warmed at 37 °C) with a parafilm membrane/cage interface for 60 min (Figure 1). The VSV-blood meal consisted of defibrinated sheep blood (Lampire Biological Products, Pipersville, PA, USA) mixed 1:1 with the viral stocks at titer concentrations of 8.2 log_10_ PFU/mL. Sheep blood used for the feeding experiments was free of VSV antibodies. Oral infections were performed in two biological replicates. After blood-feeding, fully engorged females were sorted from unfed and partially fed and placed in cardboard maintenance cages.

Orally and intrathoracically infected midges were maintained in environmental chambers at 25 ± 1 °C and 70–80% relative humidity with a 13:11 light: dark cycle and offered 10% sucrose solution *ad libitum*. Ten midges injected with each VSV lineage were collected at 3- and 10-days post-injection (dpi) in either 300 µL of TRIzol (Invitrogen; Thermo Fisher Scientific, Inc., Waltham, MA, USA) or 500 µL of antibiotic medium [28,37] and stored at −80 °C until further processing for RT-qPCR and virus isolation, respectively. To test midgut infection (decapitated bodies) and dissemination to salivary glands (heads with salivary glands), 60 midges infected orally with each lineage were collected at 7- and 10-days post-feeding (dpf) in 300 µL of TRIzol or 500 µL of antibiotic medium and stored at −80 °C until further processing for RT-qPCR and virus isolation, respectively.

### 2.5. RNA Extraction and RT-qPCR for Detection of VSV

Frozen midge samples in TRIzol were thawed on ice, two 2.4 mm stainless steel beads (Omni Inc., Kennesaw, GA, USA) were added, and tissues were homogenized by shaking at 3.1 m/s with a Bead Mill Homogenizer (Omni Inc.). Samples were centrifuged at 12,000× *g* for 6 min to pellet debris, and total RNA extracted using TRIzol-BCP (1-bromo-3chloropropane; Thermo Fisher Life Technologies, Waltham, MA, USA) as previously described [37]. RNA extracts were analyzed using TaqMan Fast Virus 1-Step Master Mix (Applied Biosystems; Thermo Fisher Scientific, Inc.) in an RT-qPCR assay detecting VSV-NJ L segment as previously described [37,45]. Standard curves and calculation of Cycle threshold (Ct) values were carried out with the 7500 Fast Dx software (Applied Biosystems; Thermo Fisher Scientific, Inc. Waltham, MA, USA). RT-qPCR reactions with Ct ≤ 36.5 were considered positive for VSV RNA [28,37]. To account for inter-run variations and variable efficiency of each assay, a standard positive control with known ssRNA concentration was used in every RT-qPCR assay. Ct values plotted against the log_10_ of the ssRNA VSV ng and the linear regression (y = −3.30578x + 11.02683) allowed determination of viral genomic equivalents per midge [37].

### 2.6. Virus Isolation from Infected Midges

To isolate infectious virus, frozen midges stored in 500 μL antibiotic media were thawed on ice and individually homogenized as above. Samples were centrifuged at 12,000× *g* for 6 min to pellet debris. Injected midge samples were expected to have virus titers above 2 log_10_ PFU/mL (plaque assay limit of detection), so infectious virus was directly titered by standard plaque assay using 100 μL of original homogenate. To detect small amounts of infectious virus in VSV-fed samples (individual bodies and heads), 200 μL of the original homogenate was added to a monolayer of Vero cells with 85–90% confluency in 24-well plates. An additional 300 μL of media was added to each well and plates were incubated for up to five days. Observations of cytopathic effects (CPE) after two passages indicated infectious virus within that sample [28,37]. All homogenates showing positive CPE at the first passage were further analyzed to determine infectious virus titer by standard plaque assay of the remaining original sample and reported as log_10_ PFU/mL.

### 2.7. Statistical Analysis

Infection rates were calculated as the proportion of VSV+ bodies and dissemination rates were calculated as the proportion of VSV+ heads with salivary glands attached. Data from each virus isolate were pooled from the independent replicates of each experiment and tested for normality (Kolmogorov-Smirnov test). For variables following a normal distribution, unpaired t-test and two-way ANOVA with multiple comparisons (Tukey’s test) were used to compare the statistical significance of Ct values, infection, and dissemination rates. GraphPad Prism version 9 (GraphPad Software Inc., San Diego, CA, USA) was used for statistical analysis and the creation of graphs.

## 3. Results

### 3.1. In Vitro Growth

**Figure 2:**
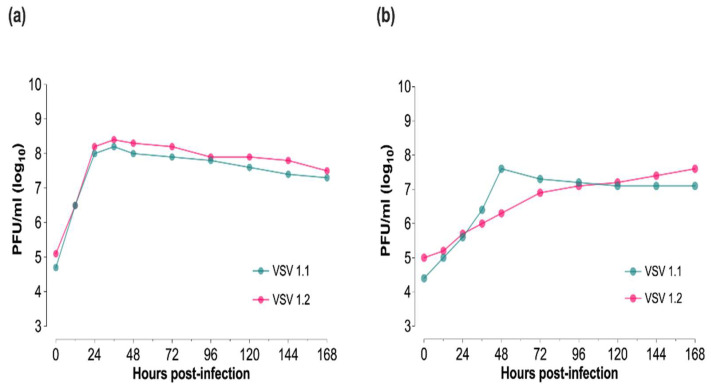
*In vitro* growth of recombinant VSV epidemic lineage 1.1 (rNJ0612NME6; teal) and the endemic lineage 1.2 (rNJ0806VCB; pink) in (**a**) porcine epithelial cells and (**b**) *Culicoides* W8 cells. All cell lines were infected at an MOI of 0.1, harvested at indicated time points, and titered by standard plaque assay in Vero cells.

To investigate the *in vitro* capacity of the BHK-derived recombinant VSV-NJ epidemic lineage 1.1 (rNJ0612NME6) and the VSV-NJ endemic lineage 1.2 (rNJ0806VCB) to replicate in target mammalian and insect vector host species, multi-step growth kinetics were evaluated in porcine epithelial and *Culicoides* cell lines, respectively (Figure 1). Overall, both viral lineages displayed similar growth kinetics in each cell line. At 36 hpi in porcine epithelial cells, VSV 1.1 and VSV 1.2 reached peak titers of 8.2 and 8.4 log_10_ PFU/mL, respectively (Figure 2a). CPE in porcine epithelial cells was observed at 48 hpi with titers remaining high in the five subsequent sample times. In *Culicoides* cells, no CPE was detected, VSV 1.1 reached peak titers of 7.6 log_10_ PFU/mL by 48 hpi and then plateaued, whereas VSV 1.2 steadily increased, reaching this same peak titer at 168 hpi (Figure 2b).

Since previous *in vivo* work was conducted in pigs [12], porcine epithelial cells were used to propagate high titer stocks of each lineage to simulate host-source viruses for subsequent midge infection studies. Whole-genome sequencing alignments revealed 100% sequence identity between rNJ0612NME or rNJ0806VCB propagated in porcine epithelial cells and their parental viruses, confirming that the change in cell source did not introduce genome changes in 1.1 and 1.2 consensus sequences.

### 3.2. VSV Intrathoracic Infection of Culicoides sonorensis Midges

**Figure 3:**
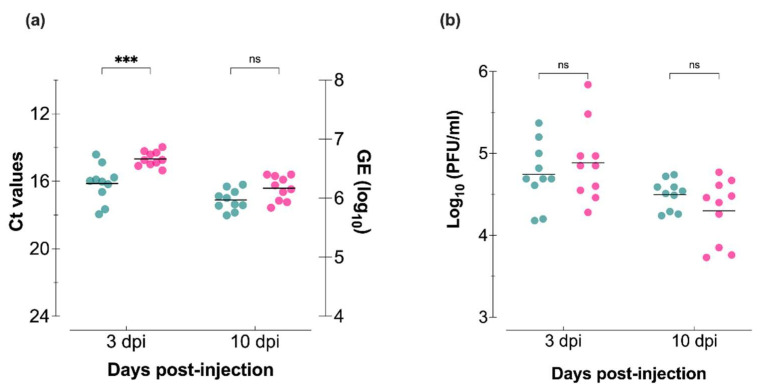
*Culicoides* midges injected with VSV epidemic lineage 1.1 (NJ0612NME6; teal) and endemic lineage 1.2 (NJ0806VCB; pink) propagated in porcine epithelial cells. (**a**) RT-qPCR cycle threshold (Ct; left Y-axis) and viral genome equivalents (GE; right Y-axis) in individual whole midges. (**b**) Infectious virus of whole-body homogenates as determined by plaque assay on Vero cells. Two-way ANOVA with multiple comparisons used to determine statistical significance as indicated (*n*= 10 midges per lineage; *p* > 0.05, ns, not significant; *** *p* < 0.001).

Intrathoracic 60 nL injections using (2.1 log_10_ PFU) were used to investigate the capacity of both VSV lineages to infect *Culicoides* tissues in the absence of a midgut barrier (Figure 1). All midge samples tested positive by RT-qPCR (Figure 3a) and plaque assay (Figure 3b). At 3 dpi, the mean values of viral RNA were significantly lower for midges injected with VSV 1.1 (*p* = 0.0002; Figure 3a); however, at 10 dpi, there were no statistically significant differences. There were also no significant differences in the infectious virus quantified by plaque assays on Vero cells at either time point (Figure 3b).

### 3.3. VSV Oral Infection of Culicoides sonorensis Midges

**Figure 4:**
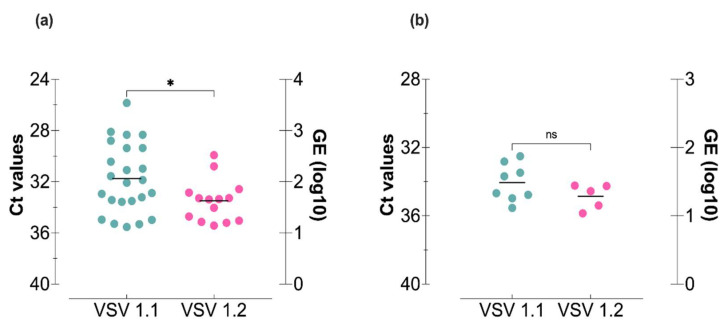
*Culicoides* midges orally infected with VSV epidemic lineage 1.1 (NJ0612NME6; teal) and the endemic lineage 1.2 (NJ0806VCB; pink) propagated in porcine epithelial cells. RT-qPCR cycle threshold (Ct; left Y-axis) and viral genome equivalents (GE; right Y-axis) in individual (**a**) bodies and (**b**) heads sampled 10 days after oral infection. Data were pooled from two biological replicates. Unpaired t-test was used to determine statistical significance as indicated (*n* = 30 midges per lineage; *p* > 0.05, ns, not significant; * *p* ≤ 0.05).

Oral infections (8.2 log_10_ PFU/mL) were used to evaluate the ability of each lineage to infect and escape the midgut barrier and then reach and infect the salivary glands for potential transmission (Figure 1). The virus titer used for the infectious blood meals was based on titers detected in oro-nasal vesicles of VSV-infected animals and was the highest titered stock virus available to ensure the highest degree of midge infection possible [1,25,45]. At 10 dpf, significantly higher values of viral RNA were detected in bodies of midges fed with the infectious meal containing VSV 1.1 (Figure 4a). Likewise, a higher percentage of bodies tested positive for viral RNA (Table 1) in midges provided with VSV 1.1 (80%) compared to midges orally infected with VSV 1.2 (46.7%). Although the quantity of VSV RNA in heads at 10 dpf was similar for both lineages (Figure 4b), a higher percentage of heads tested positive in midges fed with VSV 1.1 (26.7%) compared to VSV 1.2 (16.7%; Table 1).

To further correlate the molecular results with the detection of infectious VSV, virus isolation screening by CPE was performed at 7 and 10 dpf. Higher infection rates of midguts (decapitated bodies) and dissemination rates (heads with salivary glands) were consistently detected in midges fed with VSV 1.1 (Table 1). Due to the minute tissue sample size, virus quantification by plaque assay was only achieved for one head (4.2 log_10_ PFU/mL) and one body (4.8 log_10_ PFU/mL) of midges fed with VSV 1.1 and only one body (3.1 log_10_ PFU/mL) with no heads of midges fed with VSV 1.2.

## 4. Discussion

Multiple factors influence the complex epidemiology of VSV incursions into the U.S. [20]. Of these, viral genetic determinants may be contributing to the success of specific VSV lineages to spread beyond the endemic geographic range in Mexico and make successful incursions into the U.S. [10,11,46]. A recent example is the 2012 outbreak viruses where specific differences in the epidemic lineage (1.1) caused higher virulence in swine in comparison with the closest related endemic lineage (1.2) [12,19]. Additional biological interactions such as virus-vector interactions (transmissibility) may also be contributing to the ability of some viral lineages to escape endemic areas and successfully cause outbreaks in the U.S. Here we used a combination of well-established molecular and virological methods to better understand how virus-vector interactions may favor sporadic VSV incursions. Specifically, we compared the ability of VSV 1.1 and 1.2 to infect the vector *Culicoides sonorensis* and their respective transmission potential.

*In vitro*, different viral growth kinetics were seen between the two cell types (porcine vs. *Culicoides*) with CPE only detected in mammalian cells. This is consistent with the overall replicative characteristics of VSV in different cell lines and the ability of VSV to produce persistent, non-cytolytic infection cycles in insect cells [47,48]. No significant differences were seen with either VSV lineage within each cell line, although in *Culicoides* cells, VSV 1.1 reached its peak titer 5 days earlier than VSV 1.2. Previous *in vitro* research also showed no significant differences in growth characteristics between NJ0612NME6 and NJ0608VCB when compared in primary fetal porcine kidney cells and primary cultures of porcine macrophages [12]. However, phenotypical differences in virulence during the *in vivo* porcine infection point to the limitation of *in vitro* studies to determine phenotypic differences between wild-type viruses [12].

Although the laboratory colonization of insect vectors for multiple generations may result in a lower degree of genetic diversity, greater phenotypic similarity, and different microbiome composition to the current wild populations, colonized *Culicoides sonorensis* maintain high levels of genetic variation [49]. Moreover, colonized vector species provide controlled settings to conduct high throughput experimental studies and initial steps of hypothesis testing [50,51,52,53]. Our *in vivo* experimental designs incorporated two relevant routes of insect infection. Intrathoracic injection with relatively low titers was used to investigate the overall capacity of viruses to infect *Culicoides* tissues in the absence of a midgut escape barrier and to elucidate conspicuous replication differences between both VSV lineages. Subsequent oral infections reflected the natural route of vector infection and were therefore used to evaluate the midgut escape barrier and vector competence differences between both lineages. Moreover, virus detection in transmission-relevant organs (salivary glands) was used to indicate dissemination (midgut escape) with potential for transmission [25,28,54].

Early *Culicoides* infection (3 dpi) presented with higher RNA titers in midges injected with the epidemic lineage 1.1 than the endemic lineage 1.2. However, neither infectious virus nor RNA titers at later times (10 dpi) showed significant differences. Evaluation of midge infection by detection of viral RNA via RT-qPCR showed less variability within each viral group than the evaluation of infectious virus by plaque assay, suggesting potential alterations in the production of infectious particles in *Culicoides* tissues. Nevertheless, our results suggest that both lineages can efficiently infect *Culicoides* midges when the midgut barrier is circumvented via injection.

Differences in early replication points during the *in vitro* and *in vivo* experiments may be due to the cell source used to produce the initial viral inoculum (BHK and porcine epithelial cells, respectively). The alternating host cycles may also be virus-specific, constraining differently the ability of each viral lineage to initiate infection. However, VSV populations can successfully replicate in multiple cellular environments and apparent overall genetic fitness is not constrained by alternating cellular environments [47,55,56]. Non-genetic differences accompanied by different replicative strategies in invertebrate vectors (persistent, non-cytolytic replication) and vertebrate hosts (acute, cytolytic infection) may play a significant role in shaping VSV evolution and influence disease transmission dynamics [55].

In nature, vector-borne virus transmission depends on the ability of a virus to replicate within the vector after oral acquisition and reach the salivary glands to be transmitted during subsequent blood feeding on susceptible hosts. Our oral infection results showed that midges fed with epidemic VSV 1.1 had significantly higher infection rates and RNA titers along with a larger percentage of disseminated infections (transmission potential) than midges fed with endemic VSV 1.2. Since no differences in RNA titers were observed in intrathoracically injected midges, our oral infection results suggest a midgut barrier may have a significant impact on the ability of each lineage to be transmitted by midges.

In Dipteran vectors, the midgut epithelium is the first line of defense against viruses acquired during blood-feeding [26,27]. The transcription of innate immune genes against viruses in this anatomical region is highly dependent on RNAi, JAK-STAT, and Toll signaling pathways [26,27,31,56,57]. The JAK-STAT pathway is activated upon binding of cytokine-like unpaired ligands (Upd) to the transmembrane receptor Domeless (Dome). Moreover, antiviral RNAi response leads to induction of Vago, a cytokine-like mammalian interferon [56,57,58,59]. Likewise, the JAK-STAT pathway can also be activated by the binding of Vago [56,57]. Previous *in vitro* and *in vivo* research suggested that epidemic VSV 1.1 may be more efficient in disrupting the innate immune cytokine storm than endemic VSV 1.2 [12]. In primary swine macrophage cultures, infection with VSV 1.1 downregulated the transcription of interferon regulatory factor 7 (IRF-7) [12]. During experimental swine infection, pigs infected with epidemic VSV 1.1 produced lower levels of type-1 IFN than those infected with endemic VSV 1.2 virus [12]. While interferons do not exist in insects, Upd cytokine-like molecules activate the Dome receptor, which is an ortholog of the mammalian type I cytokine receptor [57].

Given the highly conserved nature of the innate immune molecules in metazoans, small genetic changes in VSV may not only affect virulence and innate immune cytokine responses in the mammalian host but also affect virus-vector interactions by disrupting the midge’s innate immunity during midgut invasion and subsequently influence dissemination and transmission rates. Given the limited information on genetic determinants of VSV infection and transmissibility and the conserved nature of the innate immunity in Dipteran vectors [31,57,60], it is possible that differences in viral replication seen between the endemic and epidemic viruses in midges may also occur in other VSV vector species such as black flies (*Simulium* spp.) [61,62,63,64,65,66].

There are multiple examples of small viral genetic changes altering mosquito vector competence, mainly by regulating the expression of transcription factors, ion-binding proteins, metabolic proteins, and immune pathways targeting specific regions of the virus genotypes [26,27,67,68,69,70,71,72]. Moreover, small genetic changes in bluetongue virus have also been shown to strongly affect *C. sonorensis* vector competence [73]. In Venezuelan equine encephalitis transmission, viral genetic changes in emergent lineages favor virus replication in the mammalian host and the insect vector [74]. Our results indicate that the genetics of the epidemic VSV lineage favored a disseminated infection of *Culicoides* midges and, therefore, would have been significantly more successful in spreading via vector-borne transmission than the endemic VSV 1.2 lineage. Considering our results in *Culicoides* and the previous results in swine [12], we propose that the genetic determinants of the epidemic VSV 1.1 favored the incursion of this lineage by increasing infection of and transmissibility by vectors and virulence in susceptible animal hosts.

## Figures and Tables

**Table 1:** VSV detection by CPE of orally infected *Culicoides* midges.

	VSV 1.1				VSV 1.2			
	CPE + Bodies (%)	CPE + Heads (%)	RT-qPCR + Bodies (%)	RT-qPCR + Heads (%)	CPE + Bodies (%)	CPE + Heads (%)	RT-qPCR + Bodies (%)	RT-qPCR + Heads (%)
7 dpf ^1^	15/30 (50%)	15/30 (50%)	ND ^2^	ND ^2^	9/30 (30%)	6/30 (20%)	ND ^2^	ND ^2^
10 dpf ^1^	17/30 (56.7%)	13/30 (43.3%)	24/30 (80%)	8/30 (26.7%)	11/30 (36.7%)	8/30 (26.7%)	14/30 (46.7%)	5/30 (16.7%)

^1^ VSV detected by cytopathic effect (CPE) screening of individual body and head homogenates after two passages on Vero cells. Data were pooled from two biological replicates. ^2^ ND, not determined.

## Data Availability

Raw data for figures available through Ag Data Commons within 30 months of publication.

## References

[1] Letchworth G.J., Rodriguez L.L., Del cbarrera J. (1999). Vesicular stomatitis. Vet. J..

[2] Cartwright B., Brown F. (1972). Serological relationships between different strains of vesicular stomatis virus. J. Gen. Virol..

[3] Kelley J.M., Emerson S.U., Wagner R.R. (1972). The glycoprotein of vesicular stomatitis virus is the antigen that gives rise to and reacts with neutralizing antibody. J. Virol..

[4] Seibold H.R., Sharp J.B. (1960). A revised concept of the pathological changes of the tongue in cattle with vesicular stomatitis. Am. J. Vet. Res..

[5] Chu R.M., Moore D.M., Conroy J.D. (1979). Experimental swine vesicular disease, pathology and immunofluorescence studies. Can. J. Comp. Med..

[6] U.S. Department of Agriculture Vesicular Stomatitis. https://www.aphis.usda.gov/aphis/ourfocus/animalhealth/animal-disease-information/cattle-disease-information/vesicular-stomatitis-info.

[7] Hanson R.P., Estupinan J., Castaneda J. (1968). Vesicular stomatitis in the Americas. Bull. Off. Int. Epizoot..

[8] Rainwater-Lovett K., Pauszek S.J., Kelley W.N., Rodriguez L.L. (2007). Molecular epidemiology of vesicular stomatitis New Jersey virus from the 2004–2005 US outbreak indicates a common origin with Mexican strains. J. Gen. Virol..

[9] Perez A.M., Pauszek S.J., Jimenez D., Kelley W.N., Whedbee Z., Rodriguez L.L. (2010). Spatial and phylogenetic analysis of vesicular stomatitis virus over-wintering in the United States. Prev. Vet. Med..

[10] Rodriguez L.L. (2002). Emergence and re-emergence of vesicular stomatitis in the United States. Virus Res..

[11] Velazquez-Salinas L., Pauszek S.J., Zarate S., Basurto-Alcantara F.J., Verdugo-Rodriguez A., Perez A.M., Rodriguez L.L. (2014). Phylogeographic characteristics of vesicular stomatitis New Jersey viruses circulating in Mexico from 2005 to 2011 and their relationship to epidemics in the United States. Virology.

[12] Velazquez-Salinas L., Pauszek S.J., Stenfeldt C., O’Hearn E.S., Pacheco J.M., Borca M.V., Verdugo-Rodriguez A., Arzt J., Rodriguez L.L. (2018). Increased virulence of an epidemic strain of vesicular stomatitis virus is associated with interference of the innate response in pigs. Front. Microbiol..

[13] Novella I.S., Zarate S., Metzgar D., Ebendick-Corpus B.E. (2004). Positive selection of synonymous mutations in vesicular stomatitis virus. J. Mol. Biol..

[14] Novella I.S. (2003). Contributions of vesicular stomatitis virus to the understanding of RNA virus evolution. Curr. Opin. Microbiol..

[15] Martinez I., Rodriguez L.L., Jimenez C., Pauszek S.J., Wertz G.W. (2003). Vesicular stomatitis virus glycoprotein is a determinant of pathogenesis in swine, a natural host. J. Virol..

[16] Martinez I., Wertz G.W. (2005). Biological differences between vesicular stomatitis virus Indiana and New Jersey serotype glycoproteins: Identification of amino acid residues modulating pH-dependent infectivity. J. Virol..

[17] Georgel P., Jiang Z., Kunz S., Janssen E., Mols J., Hoebe K., Bahram S., Oldstone M.B., Beutler B. (2007). Vesicular stomatitis virus glycoprotein G activates a specific antiviral Toll-like receptor 4-dependent pathway. Virology.

[18] Janelle V., Brassard F., Lapierre P., Lamarre A., Poliquin L. (2011). Mutations in the glycoprotein of vesicular stomatitis virus affect cytopathogenicity: Potential for oncolytic virotherapy. J. Virol..

[19] Velazquez-Salinas L., Pauszek S.J., Holinka L.G., Gladue D.P., Rekant S.I., Bishop E.A., Stenfeldt C., Verdugo-Rodriguez A., Borca M.V., Arzt J. (2020). A single amino acid substitution in the matrix protein (M51R) of vesicular stomatitis New Jersey virus impairs replication in cultured porcine macrophages and results in significant attenuation in pigs. Front. Microbiol..

[20] Rozo-Lopez P., Drolet B.S., Londono-Renteria B. (2018). Vesicular stomatitis virus transmission: A comparison of incriminated vectors. Insects.

[21] Borkent A., Marquardt W.C. (2005). The biting midges, the Ceratopogonidae (Diptera). Biology of Disease Vectors.

[22] Linley J.R., Davies J.B. (1971). Sandflies and tourism in Florida and the Bahamas and Caribbean area. J. Econ. Entomol..

[23] Boorman J., Lane R.P., Crosskey R.W. (1993). Biting midges (Ceratopogonidae). Medical Insects and Arachnids.

[24] Brandly C.A., Hanson R.P. (1957). Epizootiology of vesicular stomatitis. Am. J. Public Health Nations Health.

[25] Drolet B.S., Campbell C.L., Stuart M.A., Wilson W.C. (2005). Vector competence of *Culicoides sonorensis* (Diptera: Ceratopogonidae) for vesicular stomatitis virus. J. Med. Entomol..

[26] Mills M.K., Michel K., Pfannenstiel R.S., Ruder M.G., Veronesi E., Nayduch D. (2017). *Culicoides*-virus interactions: Infection barriers and possible factors underlying vector competence. Curr. Opin. Insect Sci..

[27] Kramer L.D., Ciota A.T. (2015). Dissecting vectorial capacity for mosquito-borne viruses. Curr. Opin. Virol..

[28] Rozo-Lopez P., Londono-Renteria B., Drolet B.S. (2021). Impacts of infectious dose, feeding behavior, and age of *Culicoides sonorensis* biting midges on infection dynamics of vesicular stomatitis virus. Pathogens.

[29] Conway M.J., Colpitts T.M., Fikrig E. (2014). Role of the vector in arbovirus transmission. Annu. Rev. Virol..

[30] Kramer L.D., Hardy J.L., Presser S.B., Houk E.J. (1981). Dissemination barriers for western equine encephalomyelitis virus in *Culex tarsalis* infected after ingestion of low viral doses. Am. J. Trop. Med. Hyg..

[31] Cheng G., Liu Y., Wang P., Xiao X. (2016). Mosquito defense strategies against viral infection. Trends Parasitol..

[32] Fu H., Leake C.J., Mertens P.P., Mellor P.S. (1999). The barriers to bluetongue virus infection, dissemination and transmission in the vector, *Culicoides variipennis* (Diptera: Ceratopogonidae). Arch. Virol..

[33] Mellor P.S., Booreman J., Baylis M. (2000). *Culicoides* biting midges: Their role as arbovirus vectors. Annu. Rev. Entomol..

[34] Nunamaker R.A., Perez de Leon A.A., Campbell C.C., Lonning S.M. (2000). Oral infection of *Culicoides sonorensis* (Diptera: Ceratopogonidae) by vesicular stomatitis virus. J. Med. Entomol..

[35] Perez de Leon A.A., O’Toole D., Tabachnick W.J. (2006). Infection of guinea pigs with vesicular stomatitis New Jersey virus Transmitted by *Culicoides sonorensis* (Diptera: Ceratopogonidae). J. Med. Entomol..

[36] Perez de Leon A.A., Tabachnick W.J. (2006). Transmission of vesicular stomatitis New Jersey virus to cattle by the biting midge *Culicoides sonorensis* (Diptera: Ceratopogonidae). J. Med. Entomol..

[37] Rozo-Lopez P., Londono-Renteria B., Drolet B.S. (2020). Venereal transmission of vesicular stomatitis virus by *Culicoides sonorensis* midges. Pathogens.

[38] Rozo-Lopez P., Park Y., Drolet B.S. (2022). Effect of constant temperatures on *Culicoides sonorensis* midge physiology and vesicular stomatitis virus infection. Insects.

[39] Velazquez-Salinas L., Pauszek S.J., Barrera J., Clark B.A., Borca M.V., Verdugo-Rodriguez A., Stenfeldt C., Arzt J., Rodriguez L.L. (2019). Validation of a site-specific recombination cloning technique for the rapid development of a full-length cDNA clone of a virulent field strain of vesicular stomatitis New Jersey virus. J. Virol. Methods.

[40] Buchholz U.J., Finke S., Conzelmann K.K. (1999). Generation of bovine respiratory syncytial virus (BRSV) from cDNA: BRSV NS2 is not essential for virus replication in tissue culture, and the human RSV leader region acts as a functional BRSV genome promoter. J. Virol..

[41] Velazquez-Salinas L., Pauszek S.J., Verdugo-Rodriguez A., Rodriguez L.L. (2018). Complete genome sequences of two vesicular stomatitis New Jersey viruses representing the 2012 US epidemic strain and its closest relative endemic strain from southern Mexico. Genome Announc..

[42] Palinski R.M., Bertram M.R., Vu L.T., Pauszek S.J., Hartwig E.J., Smoliga G.R., Stenfeldt C., Fish I.H., Hoang B.H., Phuong N.T. (2019). First genome sequence of foot-and-mouth disease virus serotype O sublineage Ind2001e from Southern Vietnam. Microbiol. Resour. Announc..

[43] Jones R.H., Foster N.M. (1974). Oral infection of *Culicoides variipennis* with bluetongue virus: Development of susceptible and resistant lines from a colony population. J. Med. Entomol..

[44] Nayduch D., Cohnstaedt L.W., Saski C., Lawson D., Kersey P., Fife M., Carpenter S. (2014). Studying *Culicoides* vectors of BTV in the post-genomic era: Resources, bottlenecks to progress and future directions. Virus Res..

[45] Hole K., Velazquez-Salinas L., Clavijo A. (2010). Improvement and optimization of a multiplex real-time reverse transcription polymerase chain reaction assay for the detection and typing of Vesicular stomatitis virus. J. Vet. Diagn. Investig..

[46] Rodriguez L.L., Bunch T.A., Fraire M., Llewellyn Z.N. (2000). Re-emergence of vesicular stomatitis in the western United States is associated with distinct viral genetic lineages. Virology.

[47] Zarate S., Novella I.S. (2004). Vesicular stomatitis virus evolution during alternation between persistent infection in insect cells and acute infection in mammalian cells Is dominated by the persistence phase. J. Virol..

[48] Llewellyn Z.N., Salman M.D., Pauszek S., Rodriguez L.L. (2002). Growth and molecular evolution of vesicular stomatitis serotype New Jersey in cells derived from its natural insect-host: Evidence for natural adaptation. Virus Res..

[49] Tabachnick W.J. (1990). Genetic variation in laboratory and field populations of the vector of bluetongue virus, *Culicoides variipennis* (Diptera: Ceratopogonidae). J. Med. Entomol..

[50] Mukhopadhyay J., Rangel E.F., Ghosh K., Munstermann L.E. (1997). Patterns of genetic variability in colonized strains of *Lutzomyia longipalpis* (Diptera: Psychodidae) and its consequences. Am. J. Trop. Med. Hyg..

[51] Ng’habi K.R., Lee Y., Knols B.G., Mwasheshi D., Lanzaro G.C., Ferguson H.M. (2015). Colonization of malaria vectors under semi-field conditions as a strategy for maintaining genetic and phenotypic similarity with wild populations. Malar. J..

[52] Munstermann L.E. (1994). Unexpected genetic consequences of colonization and inbreeding: Allozyme tracking in Culicidae (Diptera). Ann. Entomol. Soc. Am..

[53] Weiss B., Aksoy S. (2011). Microbiome influences on insect host vector competence. Trends Parasitol..

[54] Drolet B.S., van Rijn P., Howerth E.W., Beer M., Mertens P.P. (2015). A review of knowledge gaps and tools for orbivirus research. Vector Borne Zoonotic Dis..

[55] Coffey L.L., Vasilakis N., Brault A.C., Powers A.M., Tripet F., Weaver S.C. (2008). Arbovirus evolution *in vivo* is constrained by host alternation. Proc. Natl. Acad. Sci. USA.

[56] Paradkar P.N., Trinidad L., Voysey R., Duchemin J.B., Walker P.J. (2012). Secreted Vago restricts West Nile virus infection in Culex mosquito cells by activating the Jak-STAT pathway. Proc. Natl. Acad. Sci. USA.

[57] Elrefaey A.M.E., Hollinghurst P., Reitmayer C.M., Alphey L., Maringer K. (2021). Innate immune antagonism of mosquito-borne flaviviruses in humans and mosquitoes. Viruses.

[58] Souza-Neto J.A., Sim S., Dimopoulos G. (2009). An evolutionary conserved function of the JAK-STAT pathway in anti-dengue defense. Proc. Natl. Acad. Sci. USA.

[59] Deddouche S., Matt N., Budd A., Mueller S., Kemp C., Galiana-Arnoux D., Dostert C., Antoniewski C., Hoffmann J.A., Imler J.L. (2008). The DExD/H-box helicase Dicer-2 mediates the induction of antiviral activity in drosophila. Nat. Immunol..

[60] Kingsolver M.B., Huang Z., Hardy R.W. (2013). Insect Antiviral Innate Immunity: Pathways, Effectors, and Connections. J. Mol. Biol..

[61] Mead D.G., Gray E.W., Noblet R., Murphy M.D., Howerth E.W., Stallknecht D.E. (2004). Biological transmission of vesicular stomatitis virus (New Jersey serotype) by *Simulium vittatum* (Diptera: Simuliidae) to domestic swine (Sus scrofa). J. Med. Entomol..

[62] Mead D.G., Howerth E.W., Murphy M.D., Gray E.W., Noblet R., Stallknecht D.E. (2004). Black fly involvement in the epidemic transmission of vesicular stomatitis New Jersey virus (Rhabdoviridae: Vesiculovirus). Vector Borne Zoonotic Dis..

[63] Mead D.G., Mare C.J., Cupp E.W. (1997). Vector competence of select black fly species for vesicular stomatitis virus (New Jersey serotype). Am. J. Trop. Med. Hyg..

[64] Mead D.G., Mare C.J., Ramberg F.B. (1999). Bite transmission of vesicular stomatitis virus (New Jersey serotype) to laboratory mice by *Simulium vittatum* (Diptera: Simuliidae). J. Med. Entomol..

[65] Drolet B.S., Reeves W.K., Bennett K.E., Pauszek S.J., Bertram M.R., Rodriguez L.L. (2021). Identical viral genetic sequence found in black flies (*Simulium bivittatum*) and the equine index case of the 2006 U.S. vesicular stomatitis outbreak. Pathogens.

[66] Young K.I., Valdez F., Vaquera C., Campos C., Zhou L., Vessels H.K., Moulton J.K., Drolet B.S., Rozo-Lopez P., Pelzel-McCluskey A.M. (2021). Surveillance along the Rio Grande during the 2020 vesicular stomatitis outbreak reveals spatio-temporal dynamics of and viral RNA detection in black flies. Pathogens.

[67] Lambrechts L., Quillery E., Noel V., Richardson J.H., Jarman R.G., Scott T.W., Chevillon C. (2013). Specificity of resistance to dengue virus isolates is associated with genotypes of the mosquito antiviral gene Dicer-2. Proc. Biol. Sci..

[68] Brackney D.E., Beane J.E., Ebel G.D. (2009). RNAi targeting of West Nile virus in mosquito midguts promotes virus diversification. PLoS Pathog..

[69] Moudy R.M., Meola M.A., Morin L.L., Ebel G.D., Kramer L.D. (2007). A newly emergent genotype of West Nile virus is transmitted earlier and more efficiently by Culex mosquitoes. Am. J. Trop. Med. Hyg..

[70] Tsetsarkin K.A., Vanlandingham D.L., McGee C.E., Higgs S. (2007). A single mutation in chikungunya virus affects vector specificity and epidemic potential. PLoS Pathog..

[71] Ciota A.T., Bialosuknia S.M., Zink S.D., Brecher M., Ehrbar D.J., Morrissette M.N., Kramer L.D. (2017). Effects of Zika Virus Strain and Aedes Mosquito Species on Vector Competence. Emerg. Infect. Dis..

[72] Faizah A.N., Kobayashi D., Amoa-Bosompem M., Higa Y., Tsuda Y., Itokawa K., Miura K., Hirayama K., Sawabe K., Isawa H. (2020). Evaluating the competence of the primary vector, *Culex tritaeniorhynchus*, and the invasive mosquito species, *Aedes japonicus* japonicus, in transmitting three Japanese encephalitis virus genotypes. PLoS Negl. Trop. Dis..

[73] van Gennip R.G.P., Drolet B.S., Rozo Lopez P., Roost A.J.C., Boonstra J., van Rijn P.A. (2019). Vector competence is strongly affected by a small deletion or point mutations in bluetongue virus. Parasit. Vectors.

[74] Anishchenko M., Bowen R.A., Paessler S., Austgen L., Greene I.P., Weaver S.C. (2006). Venezuelan encephalitis emergence mediated by a phylogenetically predicted viral mutation. Proc. Natl. Acad. Sci. USA.

